# Lady Windermere Syndrome Presenting As Prolonged Febrile Illness in an Immunocompetent Woman

**DOI:** 10.7759/cureus.110224

**Published:** 2026-06-04

**Authors:** Paolo Romero Merino, Mijali L Lerzundi Mamani, Eslin Cipion-Bueno, Oscar M Herrera, Edward S Ramos Maguiña

**Affiliations:** 1 Primary Health Care Management, Valladolid Este, Valladolid, ESP; 2 Gynecology, Universidad Aquino Bolivia, Cusco, PER; 3 Health Sciences, Universidad Autónoma de Santo Domingo, Santo Domingo, DOM; 4 Orthopedics, Universidad Salvadoreña Alberto Masferrer, San Salvador, SLV; 5 Internal Medicine, Universidad Peruana Cayetano Heredia, Lima, PER

**Keywords:** bronchiectasis, febrile syndrome, immunocompetent host, lady windermere syndrome, mac lung disease, middle lobe syndrome, mycobacterium avium complex, nontuberculous mycobacteria

## Abstract

Lady Windermere syndrome is a distinct clinical entity associated with *Mycobacterium avium* complex infection that classically affects immunocompetent elderly women, particularly those with voluntary cough suppression, resulting in impaired airway clearance and mucus stasis within dependent bronchi. The condition has also been associated with a characteristic lean body habitus and thoracic skeletal abnormalities that may further compromise mucociliary clearance. We report the case of a 60-year-old woman with a history of pulmonary embolism and hypothyroidism who presented with a 45-day history of persistent nonproductive cough, intermittent fever, and right-sided chest discomfort. Physical examination revealed a lean body habitus and visible pectus excavatum. Laboratory studies demonstrated elevated inflammatory markers, and chest computed tomography showed bronchiectasis predominantly involving the middle lobe and lingula. Microbiological cultures confirmed *Mycobacterium avium* infection. The patient received a targeted 12-month multidrug regimen consisting of rifampicin, ethambutol, and azithromycin, achieving microbiological clearance and marked clinical improvement, with follow-up imaging demonstrating stable radiologic findings and sustained remission.

## Introduction

Nontuberculous mycobacteria (NTM) constitute a diverse group of environmental microorganisms that have been progressively recognized as a major cause of chronic lung disease in both immunocompromised and immunocompetent patients [1,2]. Within this group, the *Mycobacterium avium* complex (MAC) is one of the most frequently implicated pathogens. NTM lung disease often presents with nonspecific symptoms such as chronic cough, fatigue, and weight loss, which can hinder its recognition and delay diagnosis [3].

Lady Windermere syndrome (LWS) represents a particular form of MAC lung infection, typically described in elderly, thin, nonsmoking women without significant prior lung disease [4]. This condition is characterized by bronchiectasis predominantly affecting the right middle lobe and lingula, which has been associated with impaired mucociliary clearance and anatomical factors that promote secretion retention [2,4].

Additionally, a characteristic phenotype has been described in these patients, including low body mass index and chest wall abnormalities such as pectus excavatum or scoliosis, which could contribute to impaired respiratory mechanics and bronchial secretion clearance [4,5].

Despite presenting a relatively recognizable clinical pattern, LWS can manifest atypically, particularly with systemic features such as prolonged fever and minimal respiratory symptoms, posing significant diagnostic challenges and frequently leading to delayed diagnosis. It is therefore essential to consider this entity in the differential diagnosis of patients with prolonged febrile syndromes of unclear etiology, even in the absence of prominent pulmonary complaints [1,2]. Current guidelines recommend a macrolide-based multidrug regimen for the treatment of MAC pulmonary disease, with treatment duration of 12 months following culture conversion. Herein, we report an atypical presentation of Lady Windermere syndrome in a 60-year-old immunocompetent woman, in whom prolonged febrile illness was the predominant clinical manifestation.

## Case presentation

A 60-year-old woman was evaluated in the Internal Medicine department of a tertiary care hospital for a prolonged febrile syndrome associated with respiratory symptoms. Her past medical history was notable for a remote pulmonary embolism following saphenectomy, prior oophorectomy due to an ovarian abscess, and hypothyroidism diagnosed two years earlier after a previous episode of prolonged fever of unclear etiology. She was receiving levothyroxine (50 μg five days per week and 75 μg two days per week). She denied smoking, drug allergies, or chronic cardiopulmonary disease.

The patient presented with a 45-day history of intermittent high-grade fever, headache, right-sided otalgia, right shoulder pain, persistent dry cough, and right-sided chest discomfort. The cough was predominantly nonproductive, with occasional scant sputum. She had been treated empirically with amoxicillin-clavulanate without significant improvement. She denied dyspnea, gastrointestinal, or urinary symptoms but reported mild functional decline and intermittent febrile episodes.

On physical examination, she was hemodynamically stable, with blood pressure of 127/80 mmHg, heart rate of 79 beats per minute, and oxygen saturation of 94% on room air. No lymphadenopathy or thyroid enlargement was noted. Cardiac examination was normal. Pulmonary auscultation revealed preserved breath sounds with a focal rhonchus over the right hemithorax. The remainder of the examination was unremarkable.

Laboratory evaluation revealed elevated inflammatory markers alongside normal hematological and biochemical parameters. Tumor markers were within normal limits, with the exception of a mildly elevated CA-125. Autoimmune workup was unremarkable. Infectious serology demonstrated prior exposure to Epstein-Barr virus, cytomegalovirus, and *Chlamydia pneumoniae*, with a positive *Coxiella burnetii* phase II IgG titer. Tuberculin skin testing showed 10 mm induration at 48 hours. Urinalysis revealed microscopic hematuria. Relevant laboratory, microbiological, and serological findings are summarized in Table 1. 

**Table 1:** Summary of laboratory, microbiological, and serological findings

Laboratory test	Result	Reference range	Interpretation
Inflammatory markers			
Erythrocyte sedimentation rate (ESR)	74 mm/h	<20 mm/h	↑ Elevated
C-reactive protein (CRP) - on admission	101 mg/L	<5 mg/L	↑ Markedly elevated
C-reactive protein (CRP) - at evaluation	11 mg/L	<5 mg/L	↑ Elevated
C-Reactive Protein (CRP) - follow-up	2 mg/L	<5 mg/L	Normal
ESR - follow-up	21 mm/h	<20 mm/h	Normal
Complete blood count (CBC)			
White blood cell count (WBC)	Normal	4.2–9.1 ×10³/μL	Normal
Hemoglobin	Normal	12–16 g/dL	Normal
Platelet count	Normal	150–400 ×10³/μL	Normal
Metabolic and biochemical panel			
Renal function (creatinine, BUN)	Normal	—	Normal
Hepatic enzymes (aspartate transaminase, alanine transaminase, alkaline phosphatase)	Normal	—	Normal
Electrolytes	Normal	—	Normal
Creatine kinase (CK)	Normal	—	Normal
Lactate dehydrogenase (LDH)	Normal	—	Normal
Serum ferritin	Normal	—	Normal
Vitamin B12	Normal	—	Normal
Folate	Normal	—	Normal
Prothrombin activity	87%	70–130%	Normal
Protein electrophoresis	↑ Alpha-1 and Alpha-2 globulins	—	↑ Consistent with acute phase response
Thyroid function			
Thyroid-stimulating hormone (TSH)	4.25 μIU/mL	0.4–4.0 μIU/mL	↑ Mildly elevated
Free thyroxine (fT4)	Normal	0.8–1.8 ng/dL	Normal
Tumor markers			
Carcinoembryonic antigen (CEA)	Normal	—	Normal
CA 15-3	Normal	—	Normal
CA 19-9	Normal	—	Normal
CA-125	37.2 ng/mL	<35 ng/mL	↑ Mildly elevated; may reflect inflammatory state
Autoimmune studies			
Antinuclear antibodies (ANA)	Negative	—	No autoimmune disease identified
Infectious serology			
HIV	Negative	—	Immunocompetence supported
Influenza A/B	Negative	—	Excluded
Adenovirus	Negative	—	Excluded
Respiratory syncytial virus (RSV)	Negative	—	Excluded
Mycoplasma pneumoniae	Negative	—	Atypical pneumonia excluded
Epstein–Barr virus (EBV) IgG	Positive	—	Prior exposure; no active infection
Cytomegalovirus (CMV) IgG	Positive	—	Prior exposure; no active infection
Chlamydia pneumoniae IgG	Positive	—	Prior exposure; no active infection
Coxiella burnetii phase II IgG (Q fever)	Positive	—	Consistent with past/resolved infection
Tuberculin skin test (TST)	10 mm at 48 h	<5 mm negative	Borderline; active TB excluded by workup
Microbiological cultures			
Sputum acid-fast bacilli (AFB) smear (×3)	Negative	—	No acid-fast bacilli on direct smear
Sputum culture - Löwenstein medium (2/3 samples)	Mycobacterium avium	—	Diagnostic for NTM pulmonary infection
Bronchoaspirate - bacterial and fungal culture	Negative	—	No alternative pathogens identified
Bronchoaspirate - mycobacterial culture	Mycobacterium avium	—	Confirmed NTM infection - right middle lobe
Urinalysis			
Urinalysis	Microscopic hematuria	—	Incidental; no urological symptoms

Serologic testing revealed prior exposure to Epstein-Barr virus, cytomegalovirus, and *Chlamydia pneumoniae* (IgG positive). Testing for influenza A/B, adenovirus, HIV, mycoplasma, and respiratory syncytial virus was negative. Coxiella burnetii serology demonstrated a positive phase II IgG titer. Tuberculin skin testing showed 10 mm induration at 48 hours. Urinalysis revealed microscopic hematuria.

Microbiological evaluation included three sputum samples, all negative for acid-fast bacilli on direct smear; however, culture in Löwenstein medium yielded growth of *Mycobacterium avium* in two specimens. Fiberoptic bronchoscopy revealed no endobronchial abnormalities. Bronchoaspirate obtained from the right middle lobe was negative for bacterial and fungal cultures, but confirmed *Mycobacterium avium* on mycobacterial culture.

Chest radiography demonstrated pectus excavatum and a non-homogeneous increase in density in the right paracardiac region. Computed tomography of the chest showed mild peribronchial wall thickening with subtle prominence of bronchovascular markings and scattered centrilobular micronodules distributed bilaterally, consistent with an airway-centered inflammatory process. Bronchiectasis predominantly affecting the right middle lobe and lingula, with associated tree-in-bud opacities suggestive of mucus plugging, was identified on additional inferior sections. Apical pleural thickening with fibrocicatricial changes was also noted. No pleural effusion or significant lymphadenopathy was identified (Figure 1).

**Figure 1:**
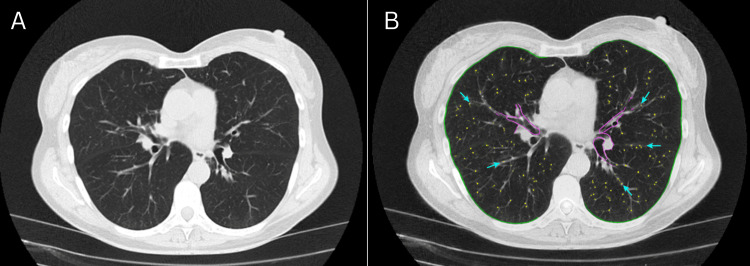
Axial CT chest images in lung window settings (A) Unannotated image. (B) Annotated image: green contour delineates pleural boundaries; magenta tracings highlight peribronchial thickening; cyan arrows indicate bronchovascular prominence; yellow dots represent centrilobular micronodules consistent with an airway-centered inflammatory process. Bronchiectasis and tree-in-bud opacities were identified on inferior sections at the level of the right middle lobe and lingula.

Computed tomography of the abdomen demonstrated incidental findings, including two small well-defined hypodense lesions in the head of the pancreas that were considered compatible with cystic lesions. A small nodular thickening was also observed in the left adrenal gland. The liver, kidneys, spleen, right adrenal gland, and gallbladder showed no relevant abnormalities.

Initially, due to the suspicion of right middle lobe inflammatory disease associated with persistent respiratory symptoms, treatment with levofloxacin was initiated, leading to partial clinical improvement. Nevertheless, the patient later experienced recurrent isolated febrile episodes accompanied by mild cough and scant sputum production. Follow-up laboratory tests demonstrated persistent inflammatory activity with a C-reactive protein level of 27 mg/L, mild leukocytosis, and an erythrocyte sedimentation rate of 72 mm/h.

Given the radiologic pattern and microbiological confirmation, a diagnosis of nodular bronchiectatic disease due to *Mycobacterium avium*, consistent with Lady Windermere syndrome, was established. Following definitive diagnosis, targeted therapy with rifampicin (600 mg daily), ethambutol (1200 mg daily), and azithromycin (500 mg daily) was initiated. Levothyroxine therapy was continued. Although current guidelines recommend a three-times-weekly regimen for noncavitary nodular bronchiectatic MAC disease, a daily regimen was selected due to the patient’s significant constitutional symptoms, prolonged febrile syndrome, elevated inflammatory markers, and radiologic disease burden at presentation. In addition, macrolide susceptibility testing results were not yet available at the time treatment was initiated, supporting a more intensive initial therapeutic approach.

During follow-up, the patient demonstrated progressive clinical improvement, with resolution of fever and respiratory symptoms. Laboratory parameters normalized, including C-reactive protein (2 mg/L) and erythrocyte sedimentation rate (21 mm/h). After microbiological conversion, antimycobacterial therapy was continued for a total of 12 months in accordance with current guidelines [2]. The patient remained asymptomatic, with stable radiologic findings and no significant adverse effects. Long-term follow-up was arranged through primary care.

## Discussion

NTM infections represent an increasingly recognized cause of chronic lung disease in adults, including immunocompetent patients [6]. The MAC complex, traditionally associated with immunosuppression, can also affect individuals without obvious immune abnormalities [7].

The diagnosis of NTM lung disease requires the presence of compatible clinical symptoms, suggestive radiological findings, and microbiological confirmation, with reasonable exclusion of other etiologies. However, the clinical presentation is often nonspecific, including chronic cough, sputum production, and fatigue, which can delay diagnosis. Furthermore, these infections frequently occur in the context of preexisting lung diseases, such as chronic obstructive pulmonary disease or bronchiectasis, which further complicates their recognition [6].

In contrast, Lady Windermere syndrome represents a distinct form of NTM lung disease that occurs in immunocompetent patients, typically middle-aged or older women who are thin, nonsmokers, and have no significant prior lung disease. This syndrome is characterized by bronchiectasis predominantly involving the right middle lobe and lingula, associated with impaired mucociliary clearance [8].

Our patient exhibited several features of the classic phenotype described for this syndrome, including female gender and absence of smoking, in addition to the presence of pectus excavatum. However, the case stands out for its atypical clinical presentation, characterized by a prolonged febrile syndrome with minimal respiratory symptoms, limited to a dry, nonproductive cough. The presence of extrapulmonary symptoms, such as headache, earache, and pain in the right shoulder, contributed to the diagnostic complexity and delayed the initial suspicion of NTM lung disease.

A mildly elevated CA-125, in the context of prolonged fever and elevated inflammatory markers, initially raised concern for occult malignancy given the patient's gynecological history. However, CA-125 elevation in the setting of systemic inflammatory and infectious processes, including mycobacterial disease, is well recognized, and this finding was ultimately attributed to the underlying inflammatory state rather than a neoplastic etiology [9].

The diagnosis in this case was based on a combination of characteristic radiological findings-bronchiectasis in the middle lobe and lingula with a tree-in-bud pattern-along with microbiological confirmation of *Mycobacterium avium*. These findings, within the appropriate clinical context, led to a diagnosis of Lady Windermere syndrome.

This case highlights the importance of considering NTM infections in immunocompetent patients with prolonged febrile illness of unclear origin, even in the absence of prominent respiratory symptoms. It also underscores the importance of recognizing the characteristic clinical phenotype of Lady Windermere syndrome to facilitate a timely diagnosis.

Treatment with rifampicin, ethambutol, and azithromycin resulted in clinical and microbiological improvement, with resolution of symptoms and normalization of inflammatory markers. Although the initial daily regimen differed from guideline-recommended intermittent therapy for noncavitary nodular bronchiectatic MAC disease [2], the patient achieved a favorable outcome without evidence of treatment failure or disease progression.

Several limitations should be acknowledged. Serum immunoglobulin levels were not obtained, precluding the formal exclusion of a subclinical humoral immunodeficiency. Nevertheless, the absence of HIV infection, recurrent opportunistic infections, or known immunosuppressive conditions, together with a reactive tuberculin skin test suggesting preserved cellular immunity, supports the likelihood that the patient was immunocompetent. Likewise, *Coxiella burnetii* phase I IgG titers were not measured, limiting the ability to definitively exclude chronic Q fever. However, the absence of endocarditis, hepatitis, vascular infection, or other clinical manifestations of chronic Q fever, combined with microbiological confirmation of *Mycobacterium avium* infection, supports the interpretation of the positive phase II IgG as reflecting prior resolved exposure rather than active disease.

In addition, macrolide susceptibility testing was not available at the time treatment was initiated. Therefore, empirical azithromycin-based therapy was selected in the absence of prior macrolide exposure and given the low likelihood of macrolide resistance. A daily treatment regimen was chosen initially because of the severity of the clinical presentation and the lack of susceptibility data, despite guideline recommendations favoring three-times-weekly therapy for this disease phenotype [2].

## Conclusions

This case illustrates that Lady Windermere syndrome may present with atypical clinical manifestations, most notably prolonged febrile illness with minimal respiratory symptoms, which can significantly delay diagnosis. This case highlights the importance of considering MAC infection in immunocompetent women with prolonged febrile syndrome of uncertain origin, even in the absence of prominent respiratory symptoms.

Recognition of the characteristic phenotype-thin build, chest wall abnormalities such as pectus excavatum, and bronchiectasis in the middle lobe and lingula, along with appropriate microbiological evaluation, is key to timely diagnosis. Nonspecific findings, such as elevated CA-125, can initially mislead the diagnostic approach and should be interpreted within the full clinical context. Combined treatment with rifampicin, ethambutol, and azithromycin for 12 months resulted in an adequate clinical and microbiological response, consistent with current recommendations.

## References

[1] Griffith DE, Aksamit T, Brown-Elliott BA (2007). An official ATS/IDSA statement: diagnosis, treatment, and prevention of nontuberculous mycobacterial diseases. Am J Respir Crit Care Med.

[2] Daley CL, Iaccarino JM, Lange C (2020). Treatment of nontuberculous mycobacterial pulmonary disease: an official ATS/ERS/ESCMID/IDSA clinical practice guideline. Clin Infect Dis.

[3] Nguyen MH, Haas MK, Kasperbauer SH (2024). Executive summary: state-of-the-art review: nontuberculous mycobacterial pulmonary disease: patients, principles, and prospects. Clin Infect Dis.

[4] Reich JM, Johnson RE (1992). Mycobacterium avium complex pulmonary disease presenting as an isolated lingular or middle lobe pattern. The Lady Windermere syndrome. Chest.

[5] Kim RD, Greenberg DE, Ehrmantraut ME (2008). Pulmonary nontuberculous mycobacterial disease: prospective study of a distinct preexisting syndrome. Am J Respir Crit Care Med.

[6] Koh WJ, Kwon OJ, Lee KS (2002). Nontuberculous mycobacterial pulmonary diseases in immunocompetent patients. Korean J Radiol.

[7] Ammar M, Ullah MS, Ahmad RU, Tanvir TH, Rehmani M (2025). The subtle evolution of pulmonary parenchymal changes: a case of lady windermere syndrome emerging from chronic MAC infection in an immunocompetent patient with scoliosis. Radiol Case Rep.

[8] Parvathaneni A, Malempati SC (2023). Lady Windermere syndrome: unravelling an older lady's nightmare. Cureus.

[9] Gómez-Aldana AJ (2013). Tuberculosis peritoneal con niveles elevados de CA 125. Rev Fac Med.

